# Does uptake of specialty care affect HRQoL development in COPD patients beneficially? A difference-in-difference analysis linking claims and survey data

**DOI:** 10.1007/s10198-022-01562-7

**Published:** 2023-01-13

**Authors:** Alisa Stöber, Pavo Marijic, Christoph Kurz, Larissa Schwarzkopf, Florian Kirsch, Anja Schramm, Reiner Leidl

**Affiliations:** 1 Institute of Health Economics and Health Care Management, Helmholtz Zentrum München, Ingolstädter Landstraße 1, 85764 Neuherberg, Munich, Germany; 2 Pettenkoffer School of Public Health, Munich, Germany; 3 Institute for Medical Information Processing, Biometry, and Epidemiology (IBE), Ludwig-Maximilians-University Munich (LMU), Munich, Germany; 4 Munich Center of Health Sciences (MC-Health), Institute for Health Economics and Management, Ludwig-Maximilians-University Munich (LMU), Munich, Germany; 5 Comprehensive Pneumology Center Munich (CPC-M), Member of the German Center for Lung Research (DZL), Munich, Germany; 6 Institut Fuer Therapieforschung (IFT), Working Group Therapy and Health Services Research, Munich, Germany; 7 Service Center of Health Care Management, AOK Bayern, Regensburg, Germany

**Keywords:** EQ-5D-5L, COPD assessment test, Longitudinal study, Real-world evidence, Specialty care, Pulmonologist, I11, H43

## Abstract

**Background:**

There is an evidence gap on whether the choice of specialty care beneficially affects health-related quality of life (HRQoL) in patients with chronic obstructive pulmonary disease (COPD). This study analyzes how newly initiated pulmonologist care affects the generic and disease-specific HRQoL in COPD patients over a period of 1 year.

**Methods:**

We linked claims data with data from two survey waves to investigate the longitudinal effect of specialty care on HRQoL using linear Difference-in-Difference models based on 1:3 propensity score matched data. Generic HRQoL was operationalized by EQ-5D-5L visual analog scale (VAS), and disease-specific HRQoL by COPD assessment test (CAT). Subgroup analyses examined COPD patients with low (GOLD AB) and high (GOLD CD) exacerbation risk.

**Results:**

In contrast to routine care patients, pulmonologists’ patients (*n* = 442) experienced no significant deterioration in HRQoL (VAS − 0.0, *p* = 0.9870; CAT + 0.5, *p* = 0.0804). Models unveiled a small comparative advantage of specialty care on HRQoL (mean change: CAT − 0.8, VAS + 2.9), which was especially pronounced for GOLD AB (CAT − 0.7; VAS + 3.1).

**Conclusion:**

The uptake of pulmonologist care had a statistically significant, but not clinically relevant, beneficial impact on the development of HRQoL by slowing down overall HRQoL deterioration within 1 year. Including specialty care more appropriately in COPD management, especially at lower disease stages (GOLD AB), could thus improve patients’ health outcome.

**Supplementary Information:**

The online version contains supplementary material available at 10.1007/s10198-022-01562-7.

## Introduction

### Background

Chronic obstructive pulmonary disease (COPD) is a serious health condition elevating mortality and morbidity, which claims more than 3 million lives per year [1]. It is the third leading cause of death worldwide [1], and the burden of COPD is even expected to increase further on account of an aging population and continued exposure to COPD risk factors (e.g., smoking) [2]. This inflammatory disease is characterized by respiratory symptoms such as cough, breathlessness, and sputum production [3]. As COPD is a progressive disease with commonly irreversible airflow obstructions, cure cannot be achieved. Therefore, the treatment focus relies on enhancing COPD management to relieve the symptoms, lengthen the lifespan and, above all, maintain or even improve (health-related) quality of life. COPD management can be implemented by, among other things, structured programs such as the German disease management programs (DMPs).

The patients’ health-related quality of life (HRQoL) is one of the most important patient-reported outcomes (PROMs) and also a key outcome in the DMP [4]. It describes how patients perceive the severity of their symptoms and the subjective impact of the disease on their daily lives [5]. Regarding treatment, health care systems and specialty care in Europe vary, for example in the number of specialists and primary care physicians but also in the role of general practitioners (GPs). In many western countries, COPD patients are principally managed by GPs who serve as gate-keepers referring their patients to specialists, whereas patients in central or eastern Europe are rather managed by pulmonologists [6, 7]. In Germany’s Statutory Health Care system, patients have free choice of specialists without a mandatory GP referral. The utilization of specialty care is covered within the scope of the statutory health insurance (SHI) and does not incur any extra costs on behalf of the patients. This means that COPD patients in Germany can freely choose whether they receive their treatment from their primary care physician only or whether a pulmonologist is incorporated in disease management. Thus, the inclusion of pulmonologists in the treatment process, if at all, does not necessarily happen in a coordinated way. Nevertheless, the commonly used care path in Germany usually starts by a referral from the general practitioner to a pulmonologist driven by criteria of medical need [8]. Therefore, the choice of specialty care in COPD treatment on a regular basis or especially for certain severity groups could be a key management aspect that is not yet or rather casually discussed in COPD disease guidelines [9–11].

Therefore, this real-world data-based study within the COPD DMP aims to analyze the 1 year longitudinal impact of newly initiated pulmonologist care on generic and disease-specific HRQoL of COPD patients to identify eventual subgroups of patients in the DMP for whom management of treatment paths and, thus, program outcome could be improved.

## Methods

### Data

We used a linked real-world data set, combining survey data and pseudonymized health insurance claims data from AOK Bayern, a large SHI fund in the district of Bavaria in southern Germany. With almost 4.5 million insurants, AOK Bayern had a 40.5% share of the district’s SHI market during our study in 2017 [12]. SHI is accessible to everyone in Germany independent of age, comorbidities, or income and covers a broad range of in- and outpatient services at almost no copayment based on the principle of medical need. SHI contributions are calculated risk-independently based on the insurants’ income [13]. Nonetheless, in Germany about 10% of the population are privately insured outside the SHI system, including mainly persons with a high income level, self-employed or civil servants [14, 15].

Our initial data set contained all insurants who participated in the structured COPD DMP of AOK Bayern. This DMP is a voluntary program with comprehensive legal requirements, to which all COPD patients, who meet the inclusion criteria [16], can get enrolled. An overview of DMP inclusion criteria can be found in the supplement (see Supplementary Information (SI) Appendix, Fig. S1). The claims data included, among others, information on sociodemographic characteristics, exacerbations, hospitalizations, comorbidities, outpatient hospital care, outpatient physician visits, and medications. Additionally, DMP-specific documentation was linked to routine claims data, which enabled the inclusion of clinical factors, such as lung function measured in forced expiratory volume in 1 s (FEV_1_) and body mass index (BMI) [17, 18].

In addition, we conducted a postal survey in two waves, addressing all 49,662 COPD DMP participants. The survey addressed generic and disease-specific HRQoL, breathlessness, determined by the modified British Medical Research Council (mMRC) [19], and further sociodemographic data (see Fig. S2 for more information). A total of 14,754 (29.7%) responders participated in the first wave in November 2017 and, of those, 9232 (62.6%) participated in the second wave in November 2018.

We then linked the survey data with the DMP and claims data. A graphical overview of data sources can be found in the supplement (Fig. S3).

We excluded all patients from our analysis, who did not respond to both survey waves and who had missing or implausible values in outcomes or covariates. For example, we excluded extreme values in the lung function FEV_1_%pred. < 10 or > 150, as we considered values above/below these thresholds as not reliable, compared to a standard lung function: the FEV_1_ percent predicted median values for individuals without pulmonary diseases are about 90, and the COPD diseases classification by GOLD starts with the lowest group “I” with a FEV_1_%pred. ≤ 80 and ends in highest group “IV” with values < 30. Indeed, there were 27 participants with values below the minimum of 10 and only 37 participants had to be excluded because of values > 150. Additionally, we disregarded patients who had received specialty care in the year before the first survey wave, as we aimed to analyze the impact of newly started specialty care treatment.

The study was approved by the ethics committee of the Ludwig-Maximilians-Universität, Munich, Germany (vote no. 17- 358) and complies with the Declaration of Helsinki.

### Outcomes/health-related quality of life assessment

We aimed to analyze the HRQoL of COPD patients receiving specialty care from pulmonologists compared with patients not treated by a pulmonologist. The main outcome, HRQoL, was measured by the disease-specific COPD Assessment Test (CAT) [20] and the generic 5-level Euro-Qol 5D (EQ-5D-5L) [21, 22], as it is recommended to use both a disease-specific and a generic measure to assess the effect on HRQoL in COPD patients [23].

The EQ-5D-5L is a validated measure of generic HRQoL in COPD [22]. It consists of a valuation section with five dimensions and five problem levels each, as well as a visual analog scale (VAS). For our analysis, we used the VAS as the generic HRQoL outcome, which (in contrast to valuations by population preferences) is a fully patient-reported outcome and thus, regarding HRQoL, also patient relevant [24]. Further, it has also been demonstrated to differentiate more precisely between COPD grades than the valuation section of the EQ-5D [24]. The VAS is a scale from 0 (worst state) to 100 (best state), on which patients can indicate their current general health perception.

The CAT measures the disease-specific HRQoL with a retrograde scale from 40 (worst state) to 0 (best state) [20]. It comprises eight dimensions (such as cough, sputum, energy, and sleep) with six answer levels each (0–5). Reasons for choosing CAT over other disease-specific HRQoL instruments can be found elsewhere [17, 25].

A clinically relevant change in HRQoL is expressed by a minimum important difference (MID), which was found to be a 6.9-point change in VAS for the EQ-5D-5L [22] and a 2.0-point change in CAT [26, 27].

### Measures and covariates

To examine the impact of specialty care on HRQoL, we primarily separated patients into a treatment and a control group. Specialty care in this context is defined as any care given by a pulmonologist, which we categorized using practitioner identification codes available in the claims data.

**Fig. 1:**
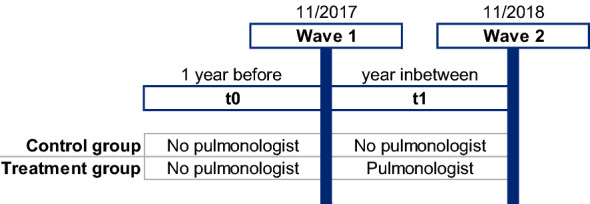
Treatment and control group

The treatment group is defined as patients who had no pulmonologist contact in the baseline year before the first survey (t0) and at least one contact with a pulmonologist in the follow-up year between surveys one and two (t1). The control group, on the other hand, comprises all patients who did not receive pulmonologist care during this observation period (see Fig. 1). In both cases, the patients may still have visited their GPs, which is why we assume that the treatment patients received combined care.

We included age, sex, exacerbation history, smoking status, lung function, mMRC, BMI group, Charlson comorbidity index, and education level as covariates (see SI Appendix, Fig. S1 for data sources). Exacerbations are adverse health events with acute worsening in respiratory symptoms [11]. The history of exacerbations (ICD-10 code J441) was based on moderate exacerbations (worsening of COPD symptoms and a doctor’s visit was required) and severe exacerbations (worsening of symptoms and a hospital stay was required) in the last 12 months before the survey [28]. Lung function was measured as FEV_1_% predicted (FEV_1_%pred.) and was condensed to an annual average wherever several measurements per year and per patient were available. The mMRC describes breathlessness on an ascending scale from 4 to 0 [19]. To calculate the weighted Charlson index, a condition was only considered present if the patient received either one inpatient diagnosis or two confirmed outpatient diagnoses within two different quarters during the year prior to the baseline assessment. This so-called M2Q-criterion is well established in claims data-based research [29]. We implemented the Charlson index using the ICD-10-based coding algorithm of Quan et at. [30] without adjustment for COPD. Further information about the weighted Charlson index conditions and secureness of the diagnosis can be found in a prior description in our research project [28]. Education levels were classified as none, basic (9 years), secondary (10 years), and higher education (12–13 years) or university degree. Reference categories were female and basic education.

We further assessed the disease severity by Global Initiative for Chronic Obstructive Lung Disease (GOLD) ABCD groups [3], based on survey data, which cannot be obtained from claims or DMP data. Here, A indicates the least severe group and D the most severe group. This classification uses a combination of COPD symptoms and exacerbation history. The symptoms differentiate between groups AC versus BD and are classified by breathlessness, where mMRC ≥ 2 identifies the more severe groups B and D. On the other hand, the exacerbation history differentiates between groups AB and CD, with an occurrence of ≥ 2 moderate or at least one severe exacerbation classifying the more severe groups C and D [3].

### Nearest neighbor propensity score matching (NN PSM)

As we found imbalance in baseline characteristics between the treatment and control group, we calculated propensity scores to balance the baseline characteristics between both groups and thereby reduce possible bias [31, 32]. Especially when the treatment and control group differ in their baseline covariates, matching is recommended to ensure accurate point estimates and better inference [33]. The matching identifies untreated (i.e., control) patients who have similar baseline characteristics to the treated participants. Based on the propensity scores, we used a nearest neighbor matching (NN PSM) with a 1:3 ratio without replacement, in which one treated patient is matched with three untreated patients. We included the previously listed covariates in the matching. The covariates for the matching were retrieved from the baseline year before treatment (t0) to ensure they were not affected by the treatment [32, 34]. Further, we applied restricted cubic splines for lung function to increase the matching quality. We then assessed the performance of the NN PSM graphically and by examining the standardized mean differences (SMDs) for all covariates. For SMDs below 0.1, we considered the groups to be well matched [31, 35]. We used the MatchIt R-Package for matching, which relies on the suggestions of Ho et al. [36].

### Statistical analysis and difference-in-difference approach

Before matching, we compared the baseline characteristics of the treatment and the control group. Therefore, we calculated counts and percentages for categorical variables as well as means and standard deviations (SD) for continuous variables. We applied independent parametric *t*-tests for continuous variables and Chi-square-tests for categorical variables. We additionally evaluated the linear absolute and relative change in HRQoL over 1 year based on paired *t*-tests.

To evaluate the impact of pulmonologist care on 1 year changes in HRQoL, we applied linear difference-in-difference (DID) models with correction for time-varying variables and cluster-robust standard errors on the propensity score matched sample. By combining matching and DID, we can use the advantages of both methods and achieve a strong quasi-experimental study design with even more robust inferences [35]. For example, the combined approach relies on weaker and more credible assumptions, and it was shown to remove time-invariant systematic differences between the intervention and control group [37, 38]. Further, it can also reduce confounding bias and increase the accuracy of the results [33, 39, 40].

After the matching, we obtained a comparable set of treated and control patients in the pre-treatment period, which is also more balanced in terms of potentially confounding baseline variables. The DID analysis enabled us to estimate the causal effect of an intervention (= pulmonologist care) on the outcome (= HRQoL) by calculating the differences in outcomes between the treatment and control groups, before and after treatment [33].

To account for dependence between observations within the matched pairs, our DID models rely on generalized linear models, and we used the coeftest function from the lmtest R-package [41] and the vcovCL function from the sandwich R-package [42, 43] to estimate the coefficients and cluster-robust standard errors [44, 45].

Our linear DID regression equation can be expressed as

1 $$ Y_{it} = \beta_{0} + \beta_{1} Group + \beta_{2} Time + \beta_{3} \left( {Group \times Time} \right) + \beta_{4} X_{it} + \varepsilon_{it} $$

$$  Group_{{it}}  = \left\{ {\begin{array}{*{20}c}    {0\,\,Control\,\,group\,\,\left( {no\,\,pulmonologist\,\,visit} \right)}  \\    {1\,\,Treatment\,\,group\,\,\left( { \ge 1\,\,pulmonologist\,\,visit} \right)}  \\   \end{array} } \right.  $$

$$  Time_{{it}} \,\, = \,\,\left\{ {\begin{array}{*{20}c}    {0\,\,Year\,\,before\,\,first\,\,survey\,\,\left( {before\,\,Nov\,\,2017} \right)}  \\    {1\,\,Year\,\,before\,\,second\,\,survey\,\,\left( {before\,\,Nov\,\,2018} \right)}  \\   \end{array} } \right.  $$

where *Y*_it_ is the outcome (HRQoL) for a patient *i* at time *t*. Whether the patient received pulmonologist care is represented by the “Group” dummy variable. The β1 coefficient accounts for the baseline differences regarding our two HRQoL outcomes (VAS and CAT) between the treatment and control group. “Time” is a dummy variable with “0” indicating the year before the first survey and “1” indicating the year between the first and second surveys. The β3 coefficient of the interaction term measures the DID estimate and thus the effect of pulmonologist care. It can be interpreted as the average treatment effect on the treated (ATT) and represents the expected HRQoL effect of pulmonologist care for COPD patients [46]. All included covariates are represented by the *X*_it_ term, while *ε*_it_ describes the error term. We included the same set of covariates in the DID model as we used in the matching before, as we assumed that they could cause variation in the DID outcome or influence selection into the treatment group [32].

The DID analysis faces two important assumptions: the “parallel trends” assumption and the “common shocks” assumption. The “parallel trends” assumption implies that the outcome for both, the treatment and the control group would change at the same rate, if no treatment had taken place [33, 46]. As we do not have multiple pre-intervention measurements of HRQoL, we cannot test the parallel trends assumption, but it was shown that prior matching can reduce possible bias [33, 35]. The “common shocks” assumption implies that all other occurrences happening within the treatment time span would affect both the treatment and the control group equally which is generally not testable [33]. A further requirement for the DID is that the composition of our treatment and control group does not change during the conduct of our study, and treatment does not “spill-over” from the treatment to the control group [33]. As the composition of the groups in our study cannot change over time by definition, a possible “selection bias across time” [35] is ruled out. A potential “selection bias across groups” [35], in which the groups themselves differ (i.e., those patients who consult a pulmonologist would differ from those who do not) is minimized by the prior matching.

To perceive differential treatment effects of specialty care on different disease severity groups, we did subgroup analysis in which we stratified the main model by GOLD groups AB and CD (because of small patient numbers in single ABCD groups).

All statistical tests were two-sided, and the significance level for *p*-values was ≤ 0.05. All analyses were performed using R version 4 [47].

### Sensitivity analysis

We performed a sensitivity analysis for all models to check the robustness of our results. Instead of propensity score matching, the sensitivity analysis used genetic matching, which is distance-based and aims to maximize the balance between the treatment and control group [48, 49]. It is a non-parametric and multivariate matching method and relies on a Mahalanobis distance matching [39, 50, 51], although it does not depend on estimating a propensity score [52]. We used a 1:3 ratio again and the same data set as in the NN PSM for the sensitivity analysis. As in the main analysis, we included all matching variables in the DID model, which should account for possible imbalance, as described by Nguyen et al. [53]. The population size argument identifying the number of random trails and the individuals used for solving the optimization problem was set to 1000.

## Results

### Study population

**Fig. 2:**
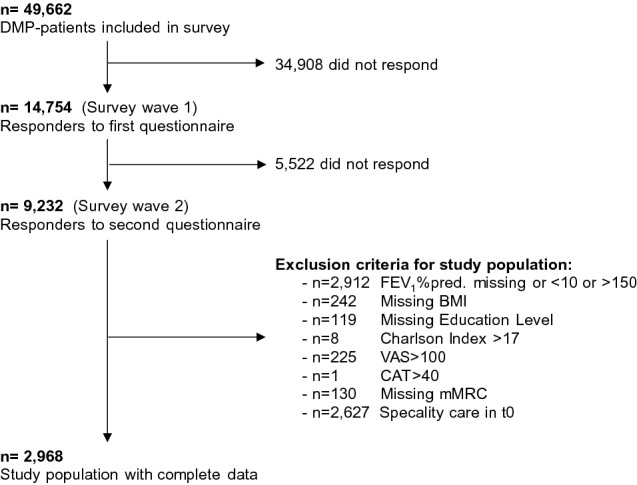
Overview of the study population. *FEV*_*1*_*%pred.* Forced expiratory volume in 1 s (percent predicted), *BMI* body mass index, *VAS* Visual Analog Scale, *CAT* COPD Assessment Test, *mMRC* Modified Medical Research Council Questionnaire

For this study, we included 2968 COPD patients. A summary of exclusion criteria is given in Fig. 2. An overview of baseline characteristics of the initial data sample is presented in the supplement (SI Appendix, Table S1).

Table 1 illustrates the baseline characteristics of the matched control and treatment group at t0. The treatment group comprised 442 patients, and the control group 2526 unmatched and 1326 matched patients respectively. On average, the treatment group had 1.6 pulmonologist visits (SD = 1.0; min = 1; max = 12). Before the matching, both groups differed significantly from each other in their baseline characteristics (see SI Appendix, Table S2). This reveals a special risk group: patients treated by a pulmonologist in the observation period tended to have not only a lower HRQoL at baseline but also a higher disease burden, as they were significantly younger with a higher number of moderate exacerbations, a lower FEV_1_%pred., and a higher mMRC compared with the unmatched control group.

**Table 1:** Baseline characteristics of treatment and control group after matching

Matched		Treatment group	Control group	*p*-value	SMD
*n*		442	1326		
Male		258 (58.4%)	775 (58.4%)	1.000	0.002
Age, years.^a^		68.46 (± 8.81)	68.72 (± 10.13)	0.629	0.027
Education^a^					
Basic (9 years.)		362 (81.9%)	1079 (81.4%)	0.977	0.038
Secondary (10 years.)		49 (11.1%)	153 (11.5%)		
Higher (12–13 years.)		12 (2.7%)	38 (2.9%)		
University		6 (1.4%)	22 (1.7%)		
None		13 (2.9%)	34 (2.6%)		
Smoking^a^					
Current		100 (22.6%)	354 (26.7%)	0.212	0.098
Ex (within last 10 years.)		112 (25.3%)	305 (23.0%)		
Never		230 (52.0%)	667 (50.3%)		
Number of Exacerbations^a^					
Moderate		0.87 (± 2.22)	0.83 (± 2.19)	0.694	0.022
Severe		0.05 (± 0.32)	0.05 (± 0.32)	0.732	0.019
FEV_1_%pred.^a^		56.68 (± 21.74)	56.79 (± 21.40)	0.929	0.005
Charlson index^a^		3.60 (± 2.76)	3.69 (± 2.65)	0.545	0.033
mMRC^b^					
0		29 (6.6%)	116 (8.7%)	0.401	0.114
1		174 (39.4%)	484 (36.5%)		
2		134 (30.3%)	389 (29.3%)		
3		95 (21.5%)	293 (22.1%)		
4		10 (2.3%)	44 (3.3%)		
BMI^a^					
< 18.5		8 (1.8%)	15 (1.1%)	0.412	0.109
≥ 18.5 to < 25		111 (25.1%)	300 (22.6%)		
≥ 25 to < 30		160 (36.2%)	482 (36.3%)		
≥ 30 to < 35		106 (24.0%)	319 (24.1%)		
≥ 35		57 (12.9%)	210 (15.8%)		
ABCD (mMRC)					
A		178 (40.3%)	512 (38.6%)	0.485	0.085
B		185 (41.9%)	590 (44.5%)		
C		25 (5.7%)	88 (6.6%)		
D		54 (12.2%)	136 (10.3%)		
HRQoL Baseline^b^					
VAS (generic)		56.62 (± 19.95)	57.85 (± 19.98)	0.264	0.061
CAT (disease-specific)		19.92 (± 7.59)	19.44 (± 7.83)	0.262	0.062

Data are presented as mean (± SD) or *n* (%). Education is represented as three German school levels by years. *P*-values based on *t*-test and Chi-square-test. Baseline data = Data previous 12 months before^a^ or from^b^ first questionnaire

*Yrs* years, *BMI* body mass index, *FEV*_*1*_*%pred.* Forced expiratory volume in 1 s (percent predicted), *mMRC* Modified Medical Research Council Questionnaire, *HRQoL* Health-related quality of life, *VAS* visual analog scale, *CAT* COPD assessment test

**Fig. 3:**
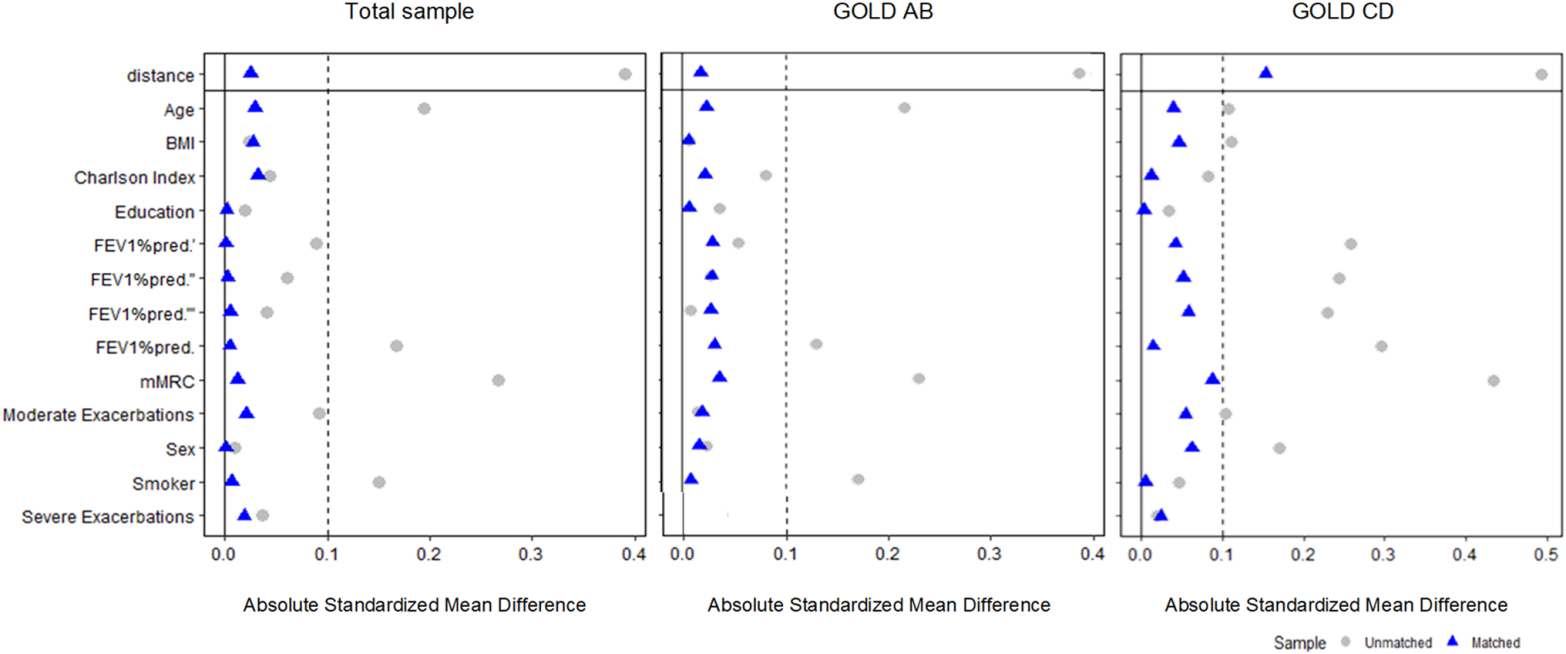
Performance of NN PSM. Performance test for GOLD AB does not include severe exacerbations because they are by definition not possible in GOLD AB. Restricted cubic splines in FEV_1_%pred. are represented by quote signs. *Yrs* years, *BMI* body mass index, *FEV*_*1*_*%pred.* forced expiratory volume in 1 s (percent predicted), *mMRC* Modified Medical Research Council Questionnaire, *HRQoL* Health-related quality of life, *VAS* visual analog scale, *CAT* COPD assessment test

The NN PSM showed a good performance with all SMDs being below 0.1 (see Fig. 3) and thus balanced the baseline characteristics between the treatment and the control group sufficiently well. In the matched treatment group, 58.4% were male, the mean age was 68.5 years, and the mean FEV_1_%pred. was 56.7. At baseline, the generic HRQoL/VAS was 56.6 (SD ± 19.9), while the disease-specific HRQoL/CAT was 19.9 (± 7.6). HRQoL baseline values for GOLD groups AB/CD can be found in SI Appendix Table S3. With regard to the ABCD groups, the baseline distribution was 40.3% (A), 41.9% (B), 5.7% (C), and 12.2% (D). Our subgroup analysis AB yielded 363 treated patients while 79 were in groups CD.

### Statistical analysis and difference-in-difference analysis

The unadjusted 1 year change in HRQoL in the unmatched data is depicted in Table 2 and SI Appendix Table S4. For the control group, we found a statistically significant overall deterioration in generic (VAS –1.3 points) and disease-specific HRQoL (CAT + 0.5 units) from t0 to t1 (see Additional file 1: Appendix, Table S4). For the treatment group, we found a similar absolute and relative change in disease-specific HRQoL (CAT + 0.5 points), which was, however, not statistically significant at the 5% level (see Table 2). On the other hand, generic HRQoL remained stable (VAS − 0.0 points).

**Table 2:** Unadjusted change in HRQoL over 1 year for treatment group (*N* = 442)

Treatment group	t0	t1	1 year change	1 year % change	*p*-value
HRQoL: VAS	56.62 (± 19.95)	56.61 (± 21.68)	− 0.01	− 0.02%	0.9870
HRQoL: CAT	19.92 (± 7.59)	20.40 (± 8.11)	0.48	2.34%	0.0804

Data are presented as mean (± SD) at baseline and follow-up for the unmatched sample

HRQoL, health-related quality of life; VAS, visual analog scale; CAT, COPD Assessment Test

**Fig. 4:**
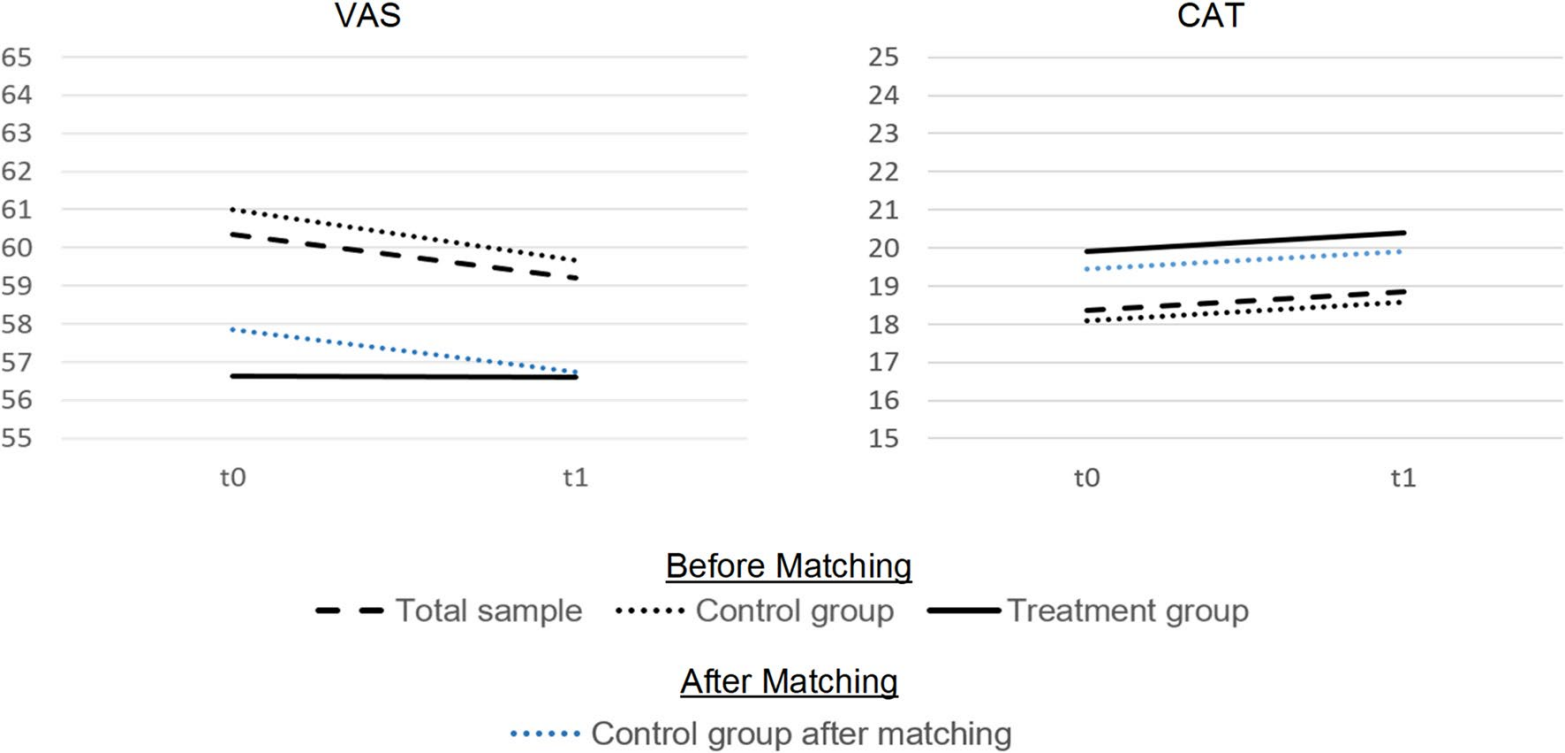
Unadjusted trends in HRQoL 1 year mean change. *VAS* Visual Analog Scale, *CAT* COPD Assessment Test

Further, we observed an overall downward trend in the unadjusted HRQoL (see Fig. 4). The mean HRQoL baseline values for patients in the treatment group were already lower at baseline (t0) than the values for patients in the control group after the 1 year follow-up (t1).

The results of the DID analysis are summarized in Table 3 and SI Appendix Table S6. The estimator (β3) for the interaction term of the dummy variables “Group” and “Time” is presented in the second column for both HRQoL outcome analyses. We observed an overall positive and significant effect of pulmonologist care on the generic HRQoL (VAS + 2.9) as well as on the disease-specific HRQoL (CAT − 0.8) for the total sample. However, this effect does not reach the MID for either VAS or CAT and is thus not clinically relevant.

**Table 3:** Results of the DID analysis of specialty treatment effect on health-related quality of life in COPD patients within 1 year after NN PSM

HRQoL outcome	Estimate	Cl.r. SE	95% CI	*p*-value	N_T_/N_C_
Model I: All matched patients of this study					
VAS	2.8490	1.0749	[0.7422, 4.9557]	0.0080***	442/1326
CAT	− 0.7805	0.3331	[− 1.4333, − 0.1277]	0.0191**	
Model II: Subgroup analysis with GOLD group AB					
VAS	3.1370	1.1559	[0.8716, 5.4025]	0.0066***	363/1089
CAT	− 0.7185	0.3578	[− 1.4198, − 0.0172]	0.0446**	
Model III: Subgroup analysis with GOLD group CD					
VAS	1.9146	2.3799	[− 2.7499, 6.5791]	0.4211	79/237
CAT	− 0.0021	0.8832	[− 1.7331, 1.7289]	0.9981	

Data presents coefficients DID interaction term for the propensity score matched sample. The *p*-values were calculated to test for the statistical significance of the estimated coefficient for the treatment effect

Control variables: Age, sex, smoking history, BMI, moderate and severe exacerbations, FEV_1_%pred., mMRC, Charlson comorbidity index and education level

*HRQoL* health-related quality of life, *VAS* visual analog scale, *CAT* COPD Assessment Test, *Cl.r. SE* cluster-robust standard error, *CI* confidence interval, *NT* number of treated, *NC* number of controls, *BMI* body mass index, *FEV*_*1*_*%pred* forced expiratory volume in 1 s (% predicted), *mMRC* Modified Medical Research Council Questionnaire

*P*-Values for statistically significant results: *p ≤ 0.10

**p ≤ 0.05

***p ≤ 0.01

Within the subgroup analysis, stratified by GOLD AB/CD groups, the specialty treatment only had a significant impact on the AB groups (VAS + 3.1 and CAT − 0.7) without reaching clinical relevance.

## Sensitivity analysis

The genetic matching revealed standardized mean differences above 0.1 in model III and thus did not perform well for CD subgroup analysis (see SI Appendix, Fig. S4). Overall, the significant impact of specialty care for the total sample was confirmed for the VAS (+ 3.0) and the CAT (− 0.7). This small effect size again does not reach a clinically relevant level. For the GOLD AB subgroup analysis, a significant, but not clinically relevant, impact was achieved for the VAS (+ 2.5) and the CAT (− 0.8) (see SI Appendix, Table S5).

## Discussion

We analyzed the impact that newly initiated specialty care had on 1 year development of generic and disease-specific HRQoL in COPD patients undergoing a DMP. Additionally, we stratified our analyses by GOLD AB/CD groups. Overall, we found a beneficial impact of pulmonologist care on the development of both HRQoL measures. Even though the statistically significant differences compared with routine care did not reach clinically relevant levels, we consider them as a positive development for COPD patients because even nuances of “feeling better” are crucial in the substantially confined HRQoL. Further, the effect size might also be larger over a longer period of time. Moreover, the overall beneficial impact further increased when focusing on GOLD group AB, and thus offers an important hint at the need to better integrate specialty care at lower stages of the disease.

This is the first large Germany-based study linking claims, DMP, and survey data to analyze the effect of specialty care on generic and disease-specific HRQoL in COPD patients. Although literature about the impact of pulmonologist care on treatments, inhalation performance, hospital readmission, costs, and survival already exists [54–57], the longitudinal association between specialty care and HRQoL has not been comprehensively investigated. This restricts the possibility of comparing our results with previous literature. Cho et al. [58] also highlighted the demand for research on the time point at which COPD patients should be referred to a pulmonologist.

By comparing those patients visiting a pulmonologist with those who did not, it became evident that they represent a special at-risk patient group. Although we looked at patients who started under the same care conditions, we only included patients who had no pulmonologist contact in the year before our observation period. The patients who subsequently received specialty care from a pulmonologist at least once in the observation period tended to be younger with lower lung function (FEV_1_%pred.), a higher number of moderate exacerbations, and more severe breathlessness (mMRC), and hence reflect a group with higher disease burden. In contrast to our findings, a Spain-based study [54] did not observe milder COPD conditions for patients without pulmonologist care, which might be at least partially explained by different access pathways to pulmonologist care in different European countries.

In our unmatched cohort, this certain pulmonologist care group also differed from those patients who solely attended general practitioners. The baseline HRQoL in pulmonologist-treated patients was already remarkably lower than the HRQoL of GP patients at 1 year follow-up.

Previous evidence from our project has already unveiled an overall downward trend of HRQoL in the total study population, which even persisted in the case of significant increases in lung function and in the absence of severe exacerbations respectively [17].

Thus, keeping this some kind of “natural course of HRQoL” in mind, the stabilization of generic HRQoL (VAS) in the specialty care group might be considered a success. Indeed, pulmonologist care apparently achieves a deceleration or maybe even a cessation in unadjusted HRQoL deterioration within just 1 year, and this for a group with quite unfavorable baseline conditions. However, this finding ought to be interpreted sensitively as, in the context of regression to the mean effects, the average rate of HRQoL decrease is generally higher for COPD patients with higher baseline HRQoL [53], which applies particularly to GP patients.

In addition, all patients in the treatment group found adequate matches in the control group, even though they represented the more severe subsample, which leads to well comparable groups for analyzing patients care needs. This in turn also indicates a potential need for speciality care in the control group.

Moreover, our study sample apparently meets the correct target group: compared to the initial DMP data set, the patients in our final study sample again tended to be younger with a higher disease burden in terms of FEV_1_%pred. and moderate exacerbations, and hence again reflect a special patient group at-risk. Therefore, we believe that the need for and up-take of speciality care is increased in this risk group—which supports our research question.

The DID models unveiled a beneficial impact of pulmonologist care on generic and disease-specific HRQoL development that is able to counteract the 1 year overall deterioration in COPD patients. One possible reason for such a comparatively positive effect on HRQoL development could be that pulmonologists’ patients are more likely to receive targeted pharmacological and non-pharmacological treatment and might be able to perform better in inhalation maneuvers [54, 55]. Moreover, combined care might raise positive synergies. For example, higher adherence to guidelines was found when specialists and GPs co-managed COPD patients, compared to GP-management alone [59]. This could lead to more patient-tailored care approaches—which could result in differences in care [60, 61] and might positively affect HRQoL. An analysis of these explanatory factors was however beyond the scope of this paper. According to our subgroup analyses, special attention should be paid to early specialty care in GOLD groups AB, i.e., those with low risk of exacerbation, as the inclusion of a pulmonologist in the patient’s therapy could improve care. Martinez et al. [62] also recently underlined the need for randomized controlled trials focusing on early-stage COPD and young patients to reduce disease progression.

Both the small sample size for GOLD CD patients and the short observation period of only 1 year should be considered when interpreting the non-significant results of this subgroup. It might be possible that the overall downward trend in HRQoL, especially in these severe groups, cannot be mitigated by pulmonologist treatment as: (a) the disease is already too far advanced; (b) the time span is too short to counteract the deterioration; or (c) the sample size in this subgroup is just too small to observe the full effect. To better understand the underlying mechanisms, further research with a bigger study population and an extended observation period with more than two time points measuring quality of life is necessary.

Even though the observed beneficial impact on HRQoL was not large and did not reach clinical relevance, pulmonologist-treated patients experienced a less substantial decline in HRQoL than GP-treated patients or even a stabilization of HRQoL. Thus, specialty care is associated with a comparative advantage compared with routine care. Moreover, this significant and non-negligible advantage in HRQoL was achieved within 1 year after the initiation of specialty care. In further research, it should be investigated whether this beneficial impact of specialty care further persists after 1 year.

Presumably, stringent and best possible early inclusion of specialty care in COPD management could also reduce the risk of future exacerbations, which in turn have been demonstrated to detrimentally affect HRQoL [63–65]. Thus, further research should analyze the effect of specialty care on exacerbations to detect whether pulmonologist care represents a promising strategy for COPD management from a clinical perspective and from a PROM-based point of view.

Another important aspect for further research could also be the impact on HRQoL of improving cooperation and communication between specialists and GPs. Recent literature shows that multidisciplinary approaches such as integrated disease management can result in a clinically relevant improvement in disease-specific HRQoL [66].

## Limitations and strengths

For this study, some limitations need to be addressed. First, the overall generalizability to COPD patients might be limited. Considering all German patients diagnosed with COPD, our population faces two selection biases. Firstly, our data originate from a large regional SHI fund. Secondly, as not all patients diagnosed with COPD participate in DMPs, we only included patients in our study participating in COPD DMP; yet this refers to both the control and the treatment group. Thus, we have information on neither patients not covered by the respective DMP nor patients covered by other SHI funds. However, all German SHI funds are legally obliged to offer DMPs for COPD patients with a comparable bundle of services. The inclusion of DMP patients could result in more conservative results and in an underestimation of the effect on HRQoL. Participation in a DMP is voluntary and may thus be linked to “good risk” patients. Also, the pulmonologist care within the DMP could be different than the care for non-DMP patients, e.g., due to better quality of care [67]. Moreover, we observed an overall drop-out bias between participants who participated in the first and second survey compared with those who did not [17]. Therefore, we assume the estimated HRQoL effects to be conservative. In addition, we considered only patients who did not die during the observation time, and thus another selection bias could be present if the mortality in the two groups is differently distributed. Further, eventual changes in measurement caused a drop-out due to lack in FEV_1_ values.

Moreover, the analyses are based on observational data and patients were not randomized. Even though we used matching to counteract this, it cannot be excluded that other causal pathways exist that influenced patients’ decisions to visit a pulmonologist, which are not reflected in our data. It is for example conceivable that rurality of residence or risk aversion might affect patients’ motivation for visiting a specialist, which could possibly lead to an underestimation of our results. Additionally, the repeated measurement of HRQoL and variables could result in regression to the mean [68]. We adjusted the DID models for baseline values to account for this issue. Next, the observation period of only 1 year is a further limitation. This time span might be too short to observe the full effect of specialty care on HRQoL. Finally, we focused on the choice between GP and specialist but did not detail the differences in treatment that could possibly impact HRQoL development.

On the other hand, our study includes unique advantages. First, the research question is novel in itself and could thus be a first step for further considerations to include pulmonologist recommendations in guidelines or for enhancing interdisciplinary COPD management. Moreover, our data set describes a large real-world setting combining claims and survey data and thus allows us to include HRQoL outcomes and to focus on GOLD ABCD groups in our subgroup analysis. This focus on GOLD ABCD groups contains another advantage, as it has been shown that these are more closely related to generic and disease-specific HRQoL than GOLD I–IV classes [69].

Another important advantage is that the effect of specialty care could be examined in relation to the choice that German patients have regarding their physician, as in other countries both patient groups are not necessarily comparable, e.g., if COPD care is strictly coordinated by GPs and pulmonologist visits are only possible under certain conditions.

## Conclusion

The uptake of specialty care revealed a small positive effect: in comparison to GP-treated patients without specialty care, pulmonologist-treated patients experienced a more favorable HRQoL development over 1 year. This beneficial impact was particularly pronounced for patients with low baseline risk of exacerbations (GOLD AB). Thus, a more appropriate inclusion of specialty care reflects a relevant aspect of patient-centered COPD management, as it provides an opportunity to improve subjective patient-relevant outcomes.

### Supplementary Information

Below is the link to the electronic supplementary material.

Supplementary file1 (DOCX 359 KB)

## Data Availability

The data sets generated and/or analyzed during the current study are not publicly available according to the data protection concept approved by the responsible data security officials and the ethics committee.

## Abbreviations

BMI: Body mass index
COPD: Chronic obstructive pulmonary disease
CAT: COPD assessment test
DID: Difference-in-difference
DMP: Disease management program
EQ-5D: Euro-Qol 5 dimension questionnaire
FEV_1_: Forced expiratory volume in 1 s
FEV_1_%pred: FEV_1_ percent predicted
GOLD: Global initiative for chronic obstructive pulmonary disease
HRQoL: Health-related quality of life
MID: Minimal important difference
MMRC: Modified British medical research council questionnaire
NN PSM: Nearest neighbor propensity score matching
PROM: Patient-reported outcome measure
SD: Standard deviation
SHI: Statutory health insurance
VAS: Visual analog scale of the Euro-Qol questionnaire
