# Supplementary material for: Does uptake of specialty care affect HRQoL development in COPD patients beneficially? A difference-in-difference analysis linking claims and survey data
Source: Eur J Health Econ. 2023 Jan 13;24(9):1561–73. doi: 10.1007/s10198-022-01562-7 (PMC10550862; doi:10.1007/s10198-022-01562-7)
Supplement: Supplementary file 1 — Supplementary file1 (DOCX 359 KB) [file 10198_2022_1562_MOESM1_ESM.docx]

# **Supplementary Information (SI) for**

# **Does uptake of specialty care affect HRQoL development in COPD patients beneficially? A difference-in-difference analysis linking claims and survey data**

**This PDF file includes:**

Figs. S1 to S2 and Tables S1 to S3

**Fig. S1** Data sources and included variables

In Germany, DMPs were introduced by a legal nationwide framework in accordance to the solidarity principle. The DMP participation requires the diagnosis of COPD and the enrollment in the DMP by a physician participating in the DMP.

The selection criteria for COPD-DMP include, that patients*:

- 1. are at least 18 years old (no further restriction of e.g. 60 years)
  2. are not enrolled in Asthma-DMP
  3. have evidence of a reduction in FEV_1_ below 80% of the target value
  4. have at least one of the following criteria:
     1. Evidence of obstruction in FEV_1_ / VC <70% after bronchodilation
     2. Evidence of an increase in airway resistance or pulmonary overinflation or a gas exchange disorder in patients with FEV_1_ / VC ≥ 70% and a radiological examination of the thoracic organs that has ruled out another disease that explains the symptoms.

*(A translation of <https://www.aok.de/gp/dmp/internistische-diagnosen/einschreibung/asthma-copd>)

**Fig. S2** Postal survey

In addition to a cover letter, the questionnaire contained the EQ-5D-5L questionnaire including the VAS scale, the COPD Assessment Test (CAT), the mMRC questionnaire and questions on socioeconomic data. Due to copyright reasons, we can only provide links and references to the HRQoL and mMRC questionnaires for this supplementary file.

1. **EQ-5D-5L and VAS:**
   <https://euroqol.org/eq-5d-instruments/eq-5d-5l-about/>

Germany (German) © 2009 EuroQol Group. EQ-5D™ is a trade mark of the EuroQol Group

1. **CAT:**
   <https://www.catestonline.org/patient-site-test-page-german-germany.html>
   CAT, COPD Assessment Test and the CAT logo are trademarks of the GSK group of companies. ©2009-2022 GSK group of companies or its licensor. All rights reserved.
2. **mMRC:**Fletcher, CM. (1960). Standardised questionnaire on respiratory symptoms: a statement prepared and approved by the MRC Committee on the Aetiology of Chronic Bronchitis (MRC breathlessness score). BMJ, 1960; 2: 1662.
3. **Socioeconomic questions:**


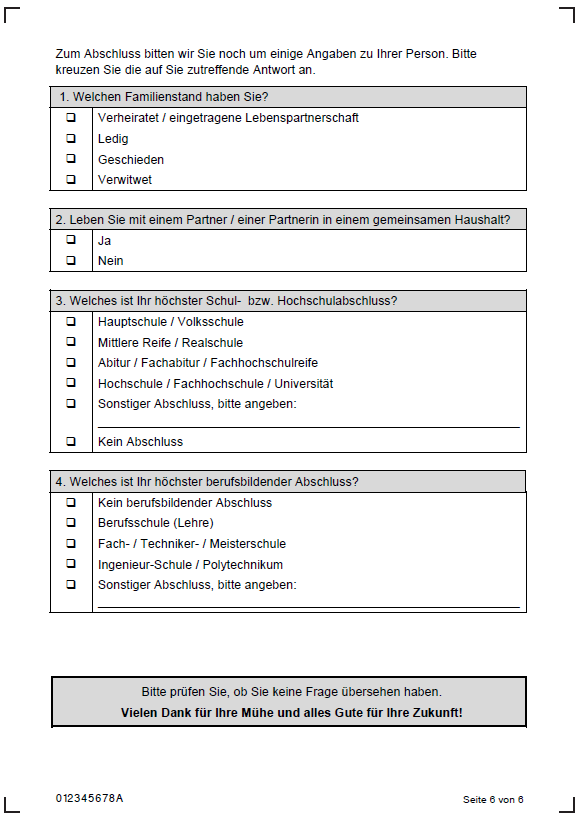


**Fig. S3** Data sources and included variables

**AOK Bayern Claims data**

- Age
- Gender
- Moderate/severe
  exacerbations
- Comorbidities
- Practitioner types

**Note:** The data linkage were subject to strict data protection regulations and ethical review.

**AOK Bayern DMP data**

**Two-wave survey**

- BMI
- Lung function (FEV_1_)
- Education
- Shortness of breath (mMRC)
- HRQoL baseline

**Fig. S4** Sensitivity analysis: Performance of Genetic Matching

**Note:** Performance test for GOLD AB does not include severe exacerbations because they are by definition not possible in GOLD AB. Restricted cubic splines in FEV_1_%pred. are represented by quote signs.

**Abbreviations:** Yrs, years; BMI, body mass index; FEV_1_%pred., forced expiratory volume in 1 second (percent predicted); mMRC, Modified Medical Research Council Questionnaire; HRQoL, Health-related quality of life; VAS, visual analog scale; CAT, COPD assessment test.

**Table S1** Baseline Characteristics of initial data set

|  |  | **Initial data set** |  | **Not in study sample** | **Study sample** |  |
| --- | --- | --- | --- | --- | --- | --- |
| n |  | 49,662 | Missings | 44,067 | 5,595 | p-value |
| Male |  | 26,939 (54.24%) |  | 23,607 (53.6%) | 3,332 (59.6%) | <0.001 |
| Age, yrs. |  | 69.40 (±11.18) | 1 | 69.44 (±11.39) | 69.06 (±9.46) | 0.016 |
| Smoker | Never | 25,976 (52.31%) |  | 22885 (51.9%) | 3,091 (55.25%) | <0.001 |
|  | Active | 13,392 (26.97%) |  | 12165 (27.6%) | 1,227 (21.93%) |  |
|  | Former | 10,294 (20.73%) |  | 9017 (20.5%) | 1,277 (22.82%) |  |
| Number of Exacerbations | Moderate | 0.77 (±1.93) |  | 0.76 (±1.91) | 0.90 (±2.09) | <0.001 |
|  | Severe | 0.09 (±0.43) |  | 0.09 (±0.43) | 0.08 (±0.44) | 0.438 |
| FEV_1_%pred. | | 57.95 (±23.77) | 13144 | 58.34 (±24.27) | 55.80 (±20.65) | <0.001 |
| Charlson index |  | 4.01 (±2.99) |  | 4.05 (±3.02) | 3.68 (±2.73) | <0.001 |
| **Notes:** Data are presented as mean (± SD) or n (%), previous 12 months before first questionnaire.  P-values based on t-test, Chi-square-test and Wilcoxon–Mann–Whitney-test. **Abbreviations:** Yrs, years; FEV_1_%pred. = Forced expiratory volume in 1 second (percent predicted). | | | | | | |

**Table S2** Baseline Characteristics of treatment and control group before matching

| **Unmatched** | | **Treatment group** | **Control group** |  |  |
| --- | --- | --- | --- | --- | --- |
| n | | 442 | 1,326 | p-value | SMD |
| Male | | 258 (58.4%) | 1,462 (57.9%) | 0.888 | 0.010 |
| Age, yrs.^a^ | | 68.46 (±8.81) | 69.87 (±10.00) | 0.006 | 0.149 |
| Education^a^ | Basic (9 yrs.) | 362 (81.9%) | 2,053 (81.3%) | 0.896 | 0.057 |
|  | Secondary (10 yrs.) | 49 (11.1%) | 281 (11.1%) |  |  |
|  | Higher (12–13 yrs.) | 12 (2.7%) | 65 (2.6%) |  |  |
|  | University | 6 (1.4%) | 53 (2.1%) |  |  |
|  | None | 13 (2.9%) | 74 (2.9%) |  |  |
| Smoking^a^ | Current  Ex (within last 10 yrs.)  Never | 100 (22.6%)  112 (25.3%)  230 (52.0%) | 614 (24.3%)  460 (18.2%)  1452 (57.5%) | 0.002 | 0.174 |
| Number of Exacerbations^a^ | Moderate | 0.87 (±2.22) | 0.67 (±1.92) | 0.045 | 0.098 |
|  | Severe | 0.05 (±0.32) | 0.04 (±0.30) | 0.452 | 0.038 |
| FEV_1_%pred.^a^ | | 56.68 (±21.74) | 60.31 (±20.48) | 0.001 | 0.172 |
| Charlson index^a^ | | 3.60 (±2.76) | 3.72 (±2.70) | 0.380 | 0.045 |
| mMRC^b^ | 0 | 29 (6.6%) | 353 (14.0%) | <0.001 | 0.297 |
|  | 1 | 174 (39.4%) | 1,095 (43.3%) |  |  |
|  | 2 | 134 (30.3%) | 632 (25.0%) |  |  |
|  | 3 | 95 (21.5%) | 396 (15.7%) |  |  |
|  | 4 | 10 (2.3%) | 50 (2.0%) |  |  |
| BMI^a^ | <18.5 | 8 (1.8%) | 27 (1.1%) | 0.159 | 0.128 |
|  | ≥18.5 to <25 | 111 (25.1%) | 525 (20.8%) |  |  |
|  | ≥25 to <30 | 160 (36.2%) | 983 (38.9%) |  |  |
|  | ≥30 to <35 | 106 (24.0%) | 622 (24.6%) |  |  |
|  | ≥35 | 57 (12.9%) | 369 (14.6%) |  |  |
| ABCD (mMRC) | A | 178 (40.3%) | 1,264 (50.0%) | <0.001 | 0.250 |
|  | B | 185 (41.9%) | 895 (35.4%) |  |  |
|  | C | 25 (5.7%) | 184 (7.3%) |  |  |
|  | D | 54 (12.2%) | 183 (7.2%) |  |  |
| HRQoL Baseline^b^ | VAS (generic) | 56.62 (±19.95) | 61.00 (±20.01) | <0.001 | 0.219 |
|  | CAT (disease-specific) | 19.92 (±7.59) | 18.09 (±7.75) | <0.001 | 0.238 |

**Notes:** Data are presented as mean (± SD) or n (%). Education is represented as three German school levels by years. P-values based on t-test and Chi-square-test. Baseline data = Data previous 12 months before^a^ or from^b^ first questionnaire.

**Abbreviations:** Yrs, years; BMI, body mass index; FEV_1_%pred., Forced expiratory volume in 1 second (percent predicted); mMRC, Modified Medical Research Council Questionnaire; HRQoL, Health-related quality of life; VAS, visual analog scale; CAT, COPD assessment test.

**Table S3** HRQoL Baseline Characteristics of treatment and control group after matching

**Table S4** Unadjusted change in HRQoL over 1 year for control group (N=1,326)

**Note:** Data are presented as mean (± SD) at baseline and follow-up for the unmatched sample.

**Abbreviations:** HRQoL, health-related quality of life; VAS, visual analog scale; CAT, COPD Assessment Test.

**Table S5** Sensitivity analysis: Results of the DID analysis of specialty treatment effect on health-related quality of life in COPD patients within 1 year after genetic matching

| HRQoL Outcome | Estimate | Cl.r. SE | 95%CI | p-value | N_T_ / N_C_ |
| --- | --- | --- | --- | --- | --- |
|  |  |  |  |  |  |
| *Model I: All matched patients of this study* | | |  |  |  |
| VAS | 3.0233 | 1.0223 | [1.0197, 5.0269] | 0.0031*** | 442/1,326 |
| CAT | –0.7345 | 0.3345 | [–1.3905, –0.0793] | 0.0280** |  |
|  |  |  |  |  |  |
| *Model II: Subgroup analysis with GOLD group AB* | | | |  |  |
| VAS | 2.51373 | 1.1400 | [0.2793, 4.7482] | 0.0275** | 363/1,089 |
| CAT | –0.8002 | 0.3465 | [–1.4793, –0.1211] | 0.0209** |  |
|  |  |  |  |  |  |
| *Model III: Subgroup analysis with GOLD group CD* | | | |  |  |
| VAS | 2.8020 | 2.2800 | [–1.6627, 7.2668] | 0.2187 | 79/237 |
| CAT | 0.3436 | 0.8159 | [–1.2555, 1.9427] | 0.6737 |  |
|  |  |  |  |  |  |

**Note:** Data presents coefficients DID interaction term for the propensity score matched sample.

The p-values for the statistical significance of the estimated coefficient for the treatment effect.

P-Values for statistically significant results: * p≤0.10; ** p≤0.05; *** p≤0.01.

**Control variables:** Age, sex, smoking history, BMI, moderate and severe exacerbations, FEV_1_%pred., mMRC, Charlson comorbidity index and education level.

**Abbreviations:** HRQoL, health-related quality of life; VAS, visual analog scale; CAT, COPD Assessment Test; Cl.r. SE, cluster-robust standard error; CI, confidence interval; NT, number of treated; NC, number of controls; BMI, body mass index; FEV_1_%pred, forced expiratory volume in 1 second (% predicted); mMRC, Modified Medical Research Council Questionnaire.

**Table S6** All estimates of DID Model I analysis: Effect of speciality treatment on health-related quality of life in COPD patients within 1 year

|  | Estimate | Cl.r. SE | 95%CI | | p-value | NT / NC |
| --- | --- | --- | --- | --- | --- | --- |
|  |  |  |  |  |  |  |
| *Model I: All matched patients of this study* | | |  |  |  | 442/1,326 |
| **VAS** |  |  |  |  |  |  |
| Intercept | 79.1148 | 2.0611 | [75.0751, | 83.1545] | <0.0001 |  |
| **Treatment*Time** | **2.8490** | **1.0749** | **[0.7423,** | **4.9557]** | **0.0080** |  |
| Treatment | -1.3865 | 0.9485 | [-3.2455, | 0.4724] | 0.1438 |  |
| Time | -0.8219 | 0.5501 | [-1.9000, | 0.2562] | 0.1351 |  |
| Smoker | -0.7119 | 0.4450 | [-1.5841, | 0.1602] | 0.1096 |  |
| Sex | -0.2158 | 0.6976 | [-1.5830, | 1.1514] | 0.7571 |  |
| Age | 0.1405 | 0.5562 | [-0.9497, | 1.2308] | 0.8005 |  |
| Education | -0.1345 | 0.3492 | [-0.8189, | 0.5499] | 0.7000 |  |
| BMI | -0.6024 | 0.2892 | [-1.1692, | -0.0355] | 0.0373 |  |
| Moderate exacerbations | -0.0911 | 0.1547 | [-0.3944, | 0.2122] | 0.5559 |  |
| Severe exacerbations | -2.6552 | 0.6976 | [-4.0226, | -1.2879] | 0.0001 |  |
| FEV_1_%pred. | -0.0016 | 0.0162 | [-0.0334, | 0.0302] | 0.9209 |  |
| mMRC | -10.2077 | 0.3796 | [-10.9517, | -9.4638] | <0.0001 |  |
| Charlson index | -0.4776 | 0.1318 | [-0.7359, | -0.2192] | 0.0003 |  |
|  |  |  |  |  |  |  |
| **CAT** |  |  |  |  |  |  |
| Intercept | 11.6119 | 0.7742 | [10.0946, | 13.1292] | <0.0001 |  |
| **Treatment*Time** | **-0.7805** | **0.3331** | **[-1.4333,** | **-0.1277]** | **0.0192** |  |
| Treatment | 0.5206 | 0.3439 | [-0.1534, | 1.1946] | 0.1300 |  |
| Time | 0.3445 | 0.1780 | [-0.0044, | 0.6933] | 0.0530 |  |
| Smoker | 0.2805 | 0.1639 | [-0.0408, | 0.6017] | 0.0870 |  |
| Sex | -0.2150 | 0.2598 | [-0.7241, | 0.2940] | 0.4077 |  |
| Age | -0.6723 | 0.2058 | [-1.0757, | -0.2690] | 0.0011 |  |
| Education | -0.0215 | 0.1477 | [-0.3110, | 0.2681] | 0.8845 |  |
| BMI | 0.1196 | 0.1076 | [-0.0914, | 0.3305] | 0.2666 |  |
| Moderate exacerbations | 0.1395 | 0.0692 | [0.0039, | 0.2750] | 0.0437 |  |
| Severe exacerbations | 1.0067 | 0.3271 | [0.3657, | 1.6477] | 0.0021 |  |
| FEV_1_%pred. | 0.0066 | 0.0060 | [-0.0052, | 0.0183] | 0.2749 |  |
| mMRC | 4.7063 | 0.1260 | [4.4592, | 4.9533] | <0.0001 |  |
| Charlson index | 0.0729 | 0.0486 | [-0.0224, | 0.1681] | 0.1340 |  |
|  |  |  |  |  |  |  |
| **Note:** Data presents DID coefficients for the propensity score matched sample. The p-values for the statistical significance of the estimated coefficient for the treatment effect.  **Abbreviations:** VAS, visual analog scale; CAT, COPD Assessment Test; Cl.r. SE, cluster-robust standard error; CI, confidence interval; NT, number of treated; NC, number of controls; BMI, body mass index; FEV_1_%pred., forced expiratory volume in 1 second (% predicted); mMRC, Modified Medical Research Council Questionnaire. | | | | | | |
|  |  |  |  |  |  |  |
|  |  |  |  |  |  |  |
|  |  |  |  |  |  |  |
